# Multilayered complex network datasets for three supply chain network archetypes on an urban road grid

**DOI:** 10.1016/j.dib.2017.12.041

**Published:** 2017-12-21

**Authors:** Nadia M. Viljoen, Johan W. Joubert

**Affiliations:** Centre for Transport Development, Department of Industrial and Systems Engineering, University of Pretoria, 0002 South Africa

**Keywords:** Multilayered complex networks, Supply chain vulnerability, Urban road networks

## Abstract

This article presents the multilayered complex network formulation for three different supply chain network archetypes on an urban road grid and describes how 500 instances were randomly generated for each archetype. Both the supply chain network layer and the urban road network layer are directed unweighted networks. The shortest path set is calculated for each of the 1 500 experimental instances. The datasets are used to empirically explore the impact that the supply chain's dependence on the transport network has on its vulnerability in Viljoen and Joubert (2017) [1]. The datasets are publicly available on Mendeley (Joubert and Viljoen, 2017) [2].

**Specifications Table**

**Table:** 

Subject area	*Complex Network Theory, Logistics and Supply Chain Management, Transport Planning*
More specific subject area	*Multilayered Complex Networks, Supply Chain Vulnerability, Urban Road Networks*
Type of data	*Extensible Markup Language (XML) files, compressed.*
How data was acquired	*The 500 random instances for each of the three supply chain network archetypes were generated using the JUNG library in Java. The shortest path sets were calculated using Dijkstra's algorithm and the JUNG library in Java.*
Data format	*Raw*
Experimental factors	*N/A*
Experimental features	*The datasets were used as input to an iterative disruption simulation that explores the impact that a supply chain's dependence on the urban road network has on its vulnerability.*
Data source location	*Not applicable. Data are randomly generated.*
Data accessibility	*The data are available in this article and publicly on Mendeley*[2]

**Value of the data**

• The dataset can be used to empirically test a number of complex network phenomena such as targeted attack & random error, spreading and synchronisation on three supply chain network archetypes layered on the urban road grid.
• The shortest path sets open up opportunities for research pertaining to supply chain optimisation and city planning.
• The dataset is a first example of a multilayered complex network that combines a supply chain (logical layer) with an urban road network (physical layer) and can be used as a prototype for modelling other parts of the supply chain.

## 1 Data

The data accompanying this article include the Extensible Markup Language (XML) files required to construct the 1500 unique multilayered complex network instances (500 instances for each of three supply chain archetypes). Each XML file contains all the information pertaining to one specific multilayered instance. It also contains the shortest path sets calculated for each instance. All datasets are publicly available from Mendeley [2]. The detailed Document Type Definition (DTD), which contains the declarations that describes the formal acceptable structure of the XML file, is available on http://www.matsim.org/files/dtd/multilayerNetwork_v1.dtd.

## 2 Experimental design, materials and methods

### 2.1 Data definitions and mathematical formulation

The generic multilayered network formulation is based on the notation presented in [3]. This formulation is then adapted for this specific scenario where one layer represents the supply chain network and the other the urban road grid.

### 2.2 Generic multilayered formulation

The *multilayer network* is a pair M=(G,C) where $G={\{G}_{m}\mathrm{; m}\in \left\{1,\ldots ,M\}\right\}$ is a family of M individual graphs $G_{m}=(X_{m};E_{m})$ which each represent a layer of M. In the generic formulation, as presented in [1], α and β refer to layers of G such that α,β∈{1,2,…,M} and α≠β. The set of nodes in layer $G_{\alpha }$ are denoted by $X_{\alpha }=\{x_{1}^{\alpha },\ldots ,x_{N_{\alpha }}^{\alpha }\}$ where $N_{\alpha }$ is the number of nodes in $G_{\alpha }$. The edges are denoted by $E_{\alpha }\subseteq X_{\alpha }\times X_{\alpha }$. The set of interconnections between nodes in $G_{\alpha }$ and $G_{\beta }$ with α≠β are defined by

$$C=E_{\alpha ,\beta }\subseteq X_{\alpha }\times \;X_{\beta };\alpha ,\beta \in \left\{1,2,\ldots ,M\right\},\alpha \neq \beta$$

Therefore the elements of $E_{\alpha ,\beta },\alpha \neq \beta$ are *interlayer* connections while elements of $E_{\alpha }$ are the *intralayer* connections.

### 2.3 Scenario-specific formulation

As presented in [1], we adapt the generic formulation to improve readability. Indices referring to layers are superscripts instead of subscripts so as not to cause confusion with node indices. We let M=(G,C) be the multilayered network where $G=\left(G^{1K},G^{2}\right)$. The first layer $\left(G^{1K}\right)$ represents the supply chain network where the nodes represent logistics facilities and the links represent the relationships between these facilities based on the movement of freight. This is also referred to as the *logical* layer. The second layer ($G^{2}$) represents the urban road network and is thus a *physical* layer.

In $G^{1K}$, K denotes the supply chain network archetype with K∈{F,S,D} where F is the Fully Connected (FC) network, S is the Single Hub (SH) network and D is the Double Hub (DH) network. The node set of $G^{1K}$ is defined as:

$$N^{1K}=12$$

$$X^{1K}=\left\{x_{1}^{1K},\ldots ,\;x_{N^{1K}}^{1K}\right\}\forall K\in \{F,S,D\}$$

and the edges by:

$$E^{1K}=\left\{e_{\mathrm{ij}}^{1K}\right\}\forall i\in \left\{1,\ldots ,N^{1K}\right\},j\in \left\{1,\ldots ,N^{1K}\right\}\;\text{and}\;i\neq j$$

where

$e_{\mathrm{ij}}^{1K}=\left\{ \begin{array}{cc} 1, & \text{if}\;x_{i}^{1K}\text{is}\;\text{connected}\;\text{to}\;x_{j}^{1K}\\ 0, & otherwise \end{array}\right.\forall K\in \mathrm{\{F},S,\mathrm{D\}}$

**Fig. 1:**
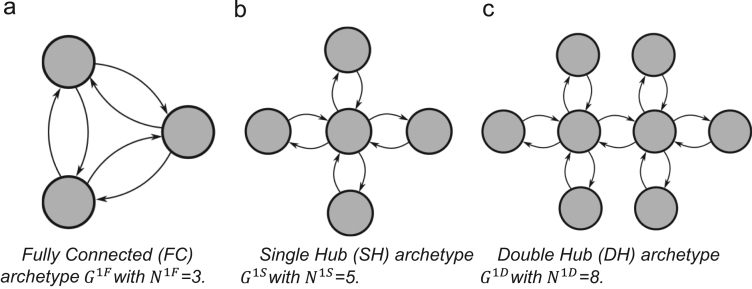
Examples of the three supply chain network archetypes (reproduced from [1]).

An FC network, $G^{1F}=\left(X^{1F},E^{1F}\right)$, assumes that all nodes in the network are directly connected to all other nodes so that each node $x_{i}^{1F}$ is directly connected to every other node $x_{j}^{1F}$ by $e_{\mathrm{ij}}^{1F}$ where i≠j. Fig. 1a) shows an example of $G^{1F}$ with three nodes. An SH network, $G^{1S}=\left(X^{1S},E^{1S}\right),$ assumes that there is one hub node with all other nodes connecting directly to the hub but not to one another as illustrated in Fig. 1b). A DH network, $G^{1D}=\left(X^{1D},E^{1D}\right)$ , assumes that there are two hub nodes with half of the remaining nodes connected directly to the first hub and the other half connected directly to the second as illustrated in Fig. 1c). $G^{1K}$ is a directed, unweighted network for all K∈{F,S,D}.

In [1] each of the supply chain network archetypes have 12 nodes (i.e. $N^{1F}=N^{1S}=N^{1D}=12$). Therefore the Single and Double Hub archetypes both have 22 edges in $\left|E^{1S}\right|$ and $\left|E^{1D}\right|$ while the Fully Connected archetype has $\left|E^{1F}\right|=N^{1F}\left(N^{1F}-1\right)=132$.

**Fig. 2:**
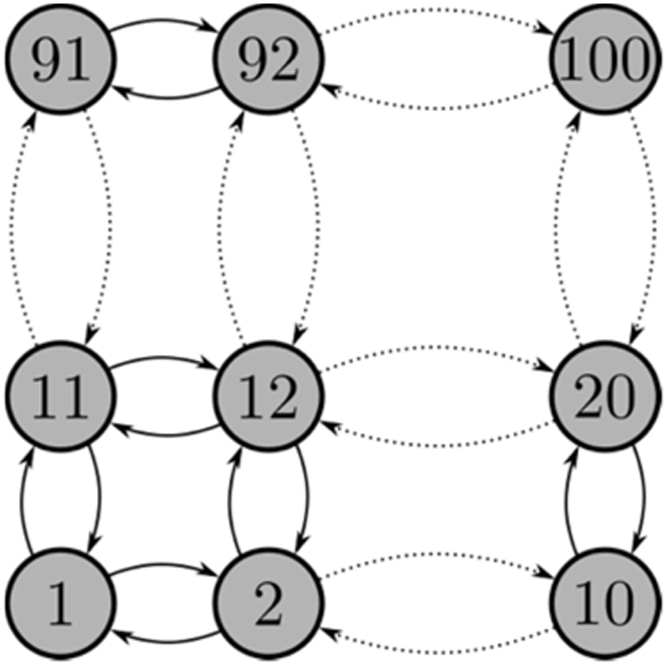
Graphic representation of the urban road grid $G^{2}$ (adapted from [1]).

$G^{2}$ represents the urban road network and is a simplified grid network with 100 intersections organised in a 10 × 10 configuration as shown in Fig. 2. The node set is defined by:

$$N^{2}=100$$

$$X^{2}=\left\{x_{1}^{2},\ldots ,\;x_{N^{2}}^{2}\right\}\forall K\in \{F,S,D\}$$

and the edges by:

$$E^{2}=\left\{e_{\mathrm{st}}^{2}\right\}\forall s,t\in \left\{1,2,\ldots ,N^{2}\right\}\text{and}\;s\neq t$$

where

$e_{\mathrm{st}}^{2}=\left\{ \begin{array}{cc} 1, & \text{if}\;x_{s}^{2}\;\text{is}\;\text{connected}\;\text{ to}\;x_{t}^{2}\\ 0, & \text{otherwise} \end{array}\right.$

This is also a directed, unweighted network and thus nodes are connected with two directed, opposite arcs, ⇆, instead of one undirected edge, ⟷. The assumption is that when a road segment in one direction fails, the associated lane in the opposite direction is not necessarily affected.

In $G^{2}=(X^{2},E^{2})$ each node $x_{s}^{2}$ is connected to four neighbours unless it is on the boundaries of the grid, in which case it is only connected to three neighbours or on the corners of the grid in which case it has only two neighbours.

#### 2.3.1 Associating the network layers

To create an instance of the multilayered network M the interlayer connections, $E^{1K,2}$, need to be defined. This is done by associating each node $x_{i}^{1K}\in X^{1K}$ with a node $x_{s}^{2}\in X^{2}$. The simplifying assumption is made that every logistics facility corresponds to the intersection closest to it. Any grid node $x_{s}^{2}$ may be associated with at most one node in $X^{1K}$. The *interlayer* adjacency matrix is denoted by $A^{\left[1K,2\right]}={(a}_{\mathrm{is}}^{1K,2})$ , where.

$a_{\mathrm{is}}^{1K,2}=\left\{ \begin{array}{cc} 1, & \;\mathrm{if}{(x}_{i}^{1K},x_{s}^{2})\in E^{1K,2}\forall i\in \{1,2,\ldots ,N^{1K}\},s\in \{1,2,\ldots ,N^{2}\}\\ 0, & \mathrm{otherwise} \end{array}\right.$

The pseudocode below (Algorithm 1) shows how the associations are randomly generated to produce $A^{\left[1F,2\right]}$.

Algorithm 1: Random generation of $A^{\left[1F,2\right]}$

**Table:** 

The algorithm used to generate $A^{\left[1S,2\right]}$ is similar and shown in the pseudocode below (Algorithm 2).

Algorithm 2: Random generation of $A^{\left[1S,2\right]}$

**Table:** 

Finally the algorithm used to generate $A^{\left[1D,2\right]}$ is slightly different as it has to constrain the assignment of nodes to hubs. The two hubs are first associated with grid nodes. Thereafter, a node is assigned to a hub if and only if the shortest path distance along $G^{2}$ from that node to the hub is less than or equal to the distance from that node to the other hub. The pseudocode below (Algorithm 3) presents the algorithm.

Algorithm 3: Random generation of $A^{[1D,2]}$

**Table:** 

The experiments conducted in [1] required large samples of M for each of the three archetypes.

We therefore generated 500 instances for each archetype with $N^{1K}=12\forall K\in \mathrm{\{F},S,\mathrm{D\}}$. We did not explicitly prevent the generation of identical $A^{\left[1K,2\right]}$ as the likelihood was negligible.

The data required to reconstruct each instance is contained in an XML file. The datafile includes the following:

1. Node set and edgelist of $G^{2};$
2. Node set and edgelist of $G^{1K}$;
3. The association between nodes in $X^{1K}$ and $X^{2}$; and
4. The shortest path sets unique to each instance (discussed in the next section).

A template of the .xml file structure is shown in Fig. 4.

#### 2.3.2 Calculating shortest path sets

The shortest path sets of an instance of Mis its unique fingerprint as it is a function of the random association of $X^{1K}$ to $X^{2}$. The characteristics of these shortest path sets are examined in [1]. Apart from the fact that these sets characterise M, they are computationally cumbersome to calculate. For these two reasons the sets are included in the input data.

**Fig. 3:**
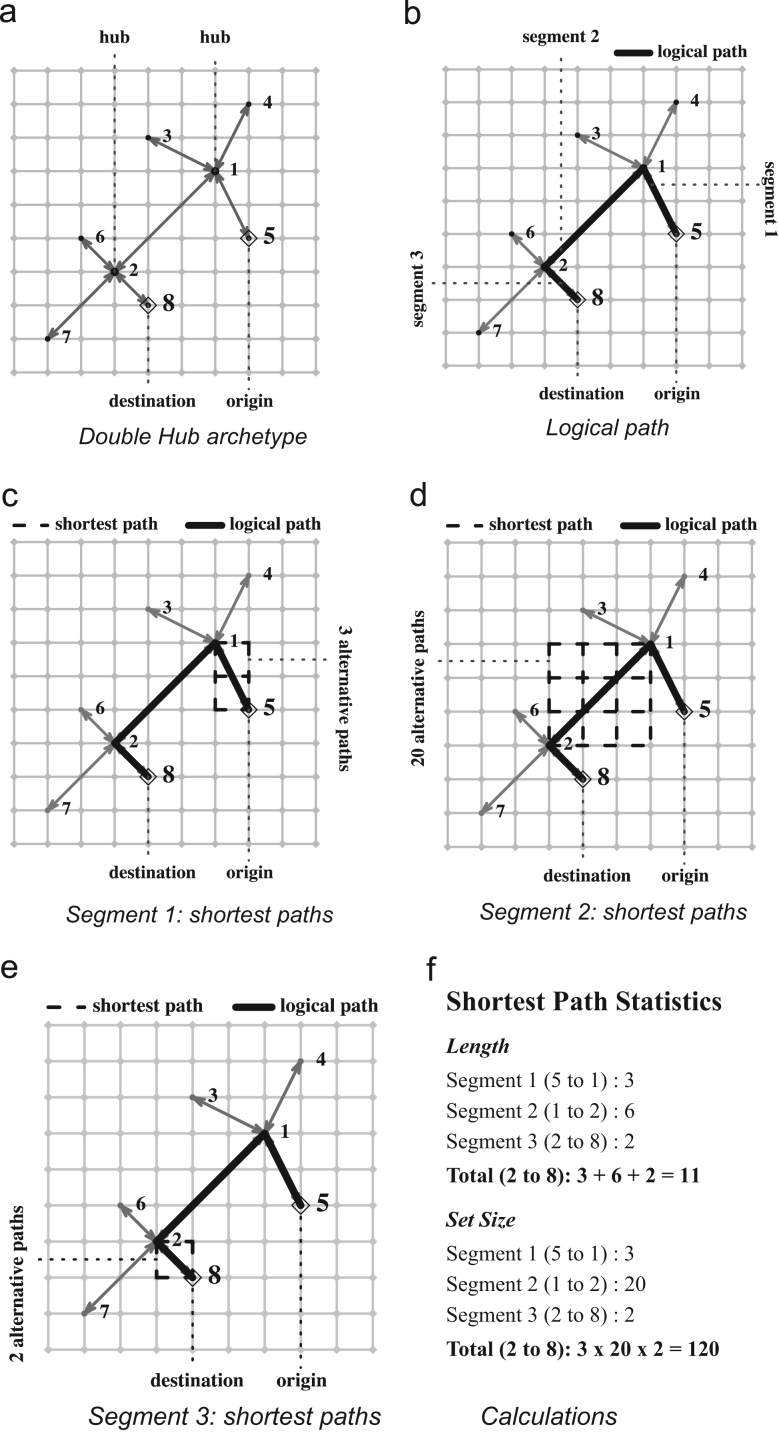
Example of calculating a shortest path set on M (reproduced from [1]).

To explain how the shortest path sets were calculated, we use the example of a Double Hub instance of M with $N^{1D}=8$. Suppose $X^{1D}$ was placed on $X^{2}$ as shown in Fig. 3a) and the shortest path had to be calculated between origin $x_{5}^{1D}$ and destination $x_{8}^{1D}$ as indicated. The logical shortest path on $G^{1D}$ consists of three segments and is highlighted in Fig. 3b). However, freight travelling between $x_{5}^{1D}$ and $x_{8}^{1D}$ must make use of the road network and is therefore also constrained by $G^{2}$. Fig. 3c) shows that there are three alternative shortest paths, each of length three, corresponding to segment one of the logical path. Similarly, there are 20 alternative paths of length six for segment two (Fig. 3d) and two alternative paths of length two for segment three (Fig. 3e). The length of the shortest path on M is the sum of the lengths of the shortest paths for each segment (3+6+2=11) while the total number of shortest paths is the product of the number of paths for each segment (3*20*2=120) (Fig. 3f). The shortest path set between $x_{5}^{1D}$ and $x_{8}^{1D}$ in M is the collection of the 120 unique path sequences and is denoted by$S_{58}$.

**Fig. 4:**
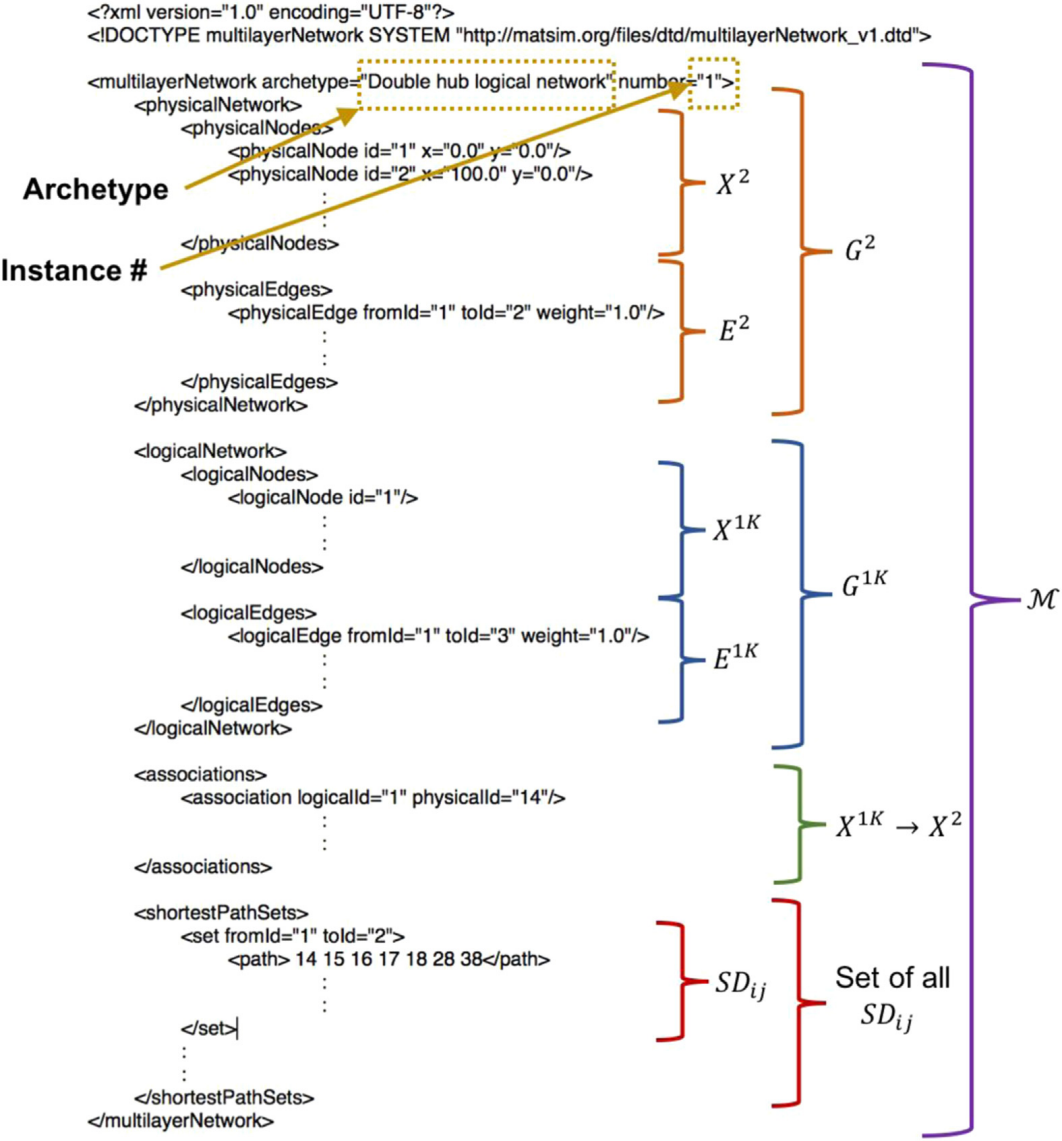
Template of the data file structure for an instance M.

Metrics pertaining to shortest path sets refer to a specific instance of M, therefore the subscripts relating to the layers and supply chain archetype are dropped for simplicity's sake. Generally we define:

$$S_{\mathrm{ij}}=\{{\mathrm{SD}}_{\mathrm{ij}},{\mathrm{SI}}_{\mathrm{ij}}\}$$

where ${\mathrm{SD}}_{\mathrm{ij}}$ is the subset of all shortest path sets between node-pairs that are directly connected in $G_{1}^{K}$ such that:

$${\mathrm{SD}}_{\mathrm{ij}}=\left\{s^{1},s^{2},\ldots ,s^{P_{\mathrm{ij}}}\right\}\;\forall \;x_{i}^{1K},x_{j}^{1K}\in E^{1K}$$

and ${\mathrm{SI}}_{\mathrm{ij}}$is the subset of all the shortest path sets between node-pairs that are indirectly connected in $G^{1K}$ such that:

$${\mathrm{SI}}_{\mathrm{ij}}=\left\{s^{1},s^{2},\ldots ,s^{P_{\mathrm{ij}}}\right\}\;\forall \;x_{i}^{1K},x_{j}^{1K}\notin E^{1K}$$

and where $P_{\mathrm{ij}}$ is the number of alternative shortest paths between any node-pair.

Enumerating and storing $S_{\mathrm{ij}}$ for all $K\in \left(F,S,D\right)\mathrm{; i},j\in \left(1,\ldots ,N^{1K}\right)\mathrm{;i}\neq j$ is too cumbersome. Instead we only calculate and store ${\mathrm{SD}}_{\mathrm{ij}}$. In the example of Fig. 3 that would mean we only calculate and store ${\mathrm{SD}}_{51}$, ${\mathrm{SD}}_{12}$ and ${\mathrm{SD}}_{28}$ but not ${\mathrm{SI}}_{58}$ as this set can always be constructed from the others when required. We calculate the shortest path sets using Dijkstra's algorithm in Java.

## References

[1] N.M. Viljoen and J.W. Joubert, Networks and Spatial Economics, 2017. 10.1007/s11067-017-9370-1.

[2] J.W. Joubert and N.M. Viljoen, Multilayer complex networks, v3. Mendeley Data, 2017. Available online from 〈http://dx.doi.org/10.17632/268byhmvv5.3〉.

[3] Boccaletti S., Bianconi G., Criado R., Del Genio C.I., Gómez-Gardeñes J., Romance M., Señdina-Nadal I., Wang Z., Zanin M. (2014). The structure and dynamics of multilayer networks. Phys. Rep..

