# Supplementary material for: Multilayered complex network datasets for three supply chain network archetypes on an urban road grid
Source: Data Brief. 2017 Dec 21;16:1069–77. doi: 10.1016/j.dib.2017.12.041 (PMC5767570; doi:10.1016/j.dib.2017.12.041)
Supplement: Supplementary file 1 — Supplementary material [file mmc1.docx]

Author declares no conflict of Interest.
